# High-intensity interval training in the prehabilitation of cancer patients—a systematic review and meta-analysis

**DOI:** 10.1007/s00520-020-05834-x

**Published:** 2020-10-26

**Authors:** Stefano Palma, Timothy Hasenoehrl, Galateja Jordakieva, Dariga Ramazanova, Richard Crevenna

**Affiliations:** 1 Department of Physical Medicine, Rehabilitation and Occupational Medicine, Medical University of Vienna, Waehringer Guertel 18-20, A-1090 Vienna, Austria; 2 Center for Medical Statistics, Informatics and Intelligent Systems (CeMSIIS), Medical University of Vienna, Vienna, Austria

**Keywords:** Prehabilitation, Preoperative care, Cancer, High-intensity interval training, Meta-analysis, Systematic review, Exercise

## Abstract

**Purpose:**

To evaluate the impact of high-intensity interval training (HIIT) on health-related outcome parameters in the prehabilitation of patients diagnosed with cancer.

**Methods:**

A systematic review and meta-analysis of comparative studies on HIIT in cancer prehabilitation conducted by screening standard databases from their inception to March 30, 2020. Outcomes of interest included cardiorespiratory fitness, feasibility, safety, clinical, and patient-reported outcomes.

**Results:**

Of the 855 identified studies, 8 articles met the inclusion criteria (7 randomized, 1 non-randomized controlled trial) with a total of 896 patients. The study protocols were heterogeneous, but the methodological quality ranged from good to high according to PEDro scale. Meta-analysis revealed a significant improvement of peak oxygen consumption (VO_2_peak) achieved with HIIT compared to usual care. Furthermore, HIIT was feasible and safe, showing low risk of adverse events and positive effects on health-related outcomes in prehabilitative settings.

**Conclusion:**

In the phase of prehabilitation, HIIT has potential health benefits in patients diagnosed with cancer and is feasible and safe to perform. Nonetheless, larger randomized controlled trials focusing on long-term effects (such as cancer recurrence or survival rates) are missing, to underline the potential relevance of HIIT for cancer patients.

**Supplementary Information:**

The online version contains supplementary material available at 10.1007/s00520-020-05834-x.

## Introduction

Cancer is estimated to have caused more than 9.8 million deaths in 2018 and is the second leading cause of death globally after cardiovascular diseases [1]. However, depending on tumor entity and stage, many highly effective individualized therapy modalities exist, leading to an increasing number of cancer survivors who require specific long-term management [2]. A substantial proportion of these patients suffer from typical adverse effects of the disease itself and its treatments (e.g., fatigue, deconditioning), causing disruption in all aspects of quality of life (QoL). Regular strength and endurance training potentially mitigates tumor- and treatment-related adverse effects and consequently have beneficial effects on QoL, physical fitness, and cancer-related fatigue [3–5]. Furthermore, exercise has been demonstrated to reduce all-cause, cancer-related, and cardiovascular disease mortality [6, 7]. Therefore, recent guidelines from the American College of Sports Medicine recommend exercise for all suitable patients with cancer, regardless of cancer stage [8]. Moreover, pooled data from European and US cohorts including more than 1.44 million participants with no cancer diagnosis at baseline indicated that higher leisure time activity levels (≥ 6 METs) resulted in a ≥ 20% risk reduction for developing esophageal, liver, lung, kidney, gastric cardia, and endometrium carcinoma compared with low activity levels, indicating that exercise intensity might also positively influence health-related outcomes [9].

Prehabilitation in oncology describes the systematic process of improving the physical, psychosocial, and nutritional status of patients between diagnosis and posttreatment recovery to increase the ability to cope with the upcoming physiological stress of the specific cancer-related therapy [10]. Presurgical training interventions in cancer patients have been the most frequently cited programs for prehabilitation, showing efficacy in reducing postoperative stress and complications, duration of hospital stay, and improving clinical outcomes by optimizing cardiopulmonary reserve prior to surgery [11–13]. Neoadjuvant therapy strategies have been shown to improve resectability of previously inoperable cancers by reducing the complexity of operations, e.g., by successfully diminishing tumor tissue [14]. However, prehabilitation programs are also targeting non-surgical cancer patients receiving pharmacological treatment only, e.g., as chemotherapy and/or radiotherapy [15–17]. In this context, a recent meta-analysis on 3257 patients with cancer indicated that moderate aerobic exercise performed at 70–80% of maximum heart rate not only was feasible and safe but also showed beneficial effects in QoL and physical functioning and maintained or at least improved fitness during concomitant chemotherapy [18]. Although not without bearing a considerably risk of bias, pooled data from another recent meta-analysis revealed that prehabilitation significantly improved mood, physical well-being, and immune function for prostate cancer patients and improved fatigue and psychological outcomes with a trend indicating better QoL among breast cancer patients [19].

In both mono- (aerobic and/or resistance exercise alone) and multimodal prehabilitative settings (exercise in combination with smoking cessation, nutritional, and psychological support), endurance exercise plays a fundamental role in the management and care of cancer patients, and increasing evidence suggests that aerobic high-intensity interval training (HIIT) may be superior to established moderate continuous intensity interventions [20]. HIIT is defined as a discontinuous mode of endurance exercise characterized by relatively short bouts of high-intensity workloads interspersed by periods of rest or low-intensity activity during recovery [21]. It was demonstrated that HIIT is effective and a safe therapy option for improving cardiovascular fitness, which is measured by peak oxygen uptake (VO_2peak_), in cancer and non-cancer patients. VO_2peak_ is strongly associated with all-cause, cancer-related, and cardiovascular disease mortality [6, 7]. The rationale behind interval training programs is that the total accumulated time of vigorous exercise is higher than what could be achieved during a single bout of continuous exercise at the same intensity before getting exhausted; moreover, it results in more pronounced cardio-metabolic adaptions than moderate intensities [22]. This time-efficient and effective method might be particularly relevant when the period from diagnosis to surgery, and therefore, the timeframe for a potential training intervention is limited. Nevertheless, patients might benefit from cardiovascular improvements, e.g., lung cancer patients awaiting lung resection surgery [23, 24]. In this regard, VO_2peak_ has emerged as the strongest independent predictor for surgical complications and survival rates in non-small cell lung cancer [25, 26]. Adams and colleagues found that HIIT increased muscular function and significantly reduced dyspnea and fatigue symptoms in testicular cancer patients. In addition, an optimization of body composition (which goes hand in hand with a reduction of the cardiovascular risk profile by reducing body fat and increasing lean mass) as well as a reduction of arterial stiffness and thickness, microvascular inflammation and dyslipidemia have been reported in this context [27]. Additionally, studies indicated that HIIT improved not only QoL but also mood state [28], emotion, pain [29], and cognitive health [30] in different study populations.

In 2019, a meta-analysis focusing on treatment and aftercare in cancer survivors revealed that HIIT significantly increased cardiorespiratory fitness in cancer patients compared with usual care (UC) [31]. However, the study did not focus specifically on outcomes of HIIT in prehabilitation. Therefore, to underline its relevance in this field, this systematic review and meta-analysis aimed to evaluate the feasibility and safety of HIIT and its impact on cardiorespiratory fitness and patient-reported outcomes compared with UC in cancer patients.

## Methods

A comprehensive systematic search of the literature was conducted by two authors in line with the Preferred Reporting Items for Systematic Review and Meta-Analysis (PRISMA) guidelines [32]; the electronic databases CENTRAL, Medline/Pubmed, Embase, and CINAHL were searched. VO_2peak_ and peak power output measured in Watts were the primary outcomes.

### Eligibility criteria

#### Inclusion criteria

- Randomized and non-randomized controlled exercise intervention trials with at least one treatment arm being HIIT (regardless of combination with other exercise intervention like resistance training [RT] and/or moderate intensity continuous training [MICT])
- Control group receives UC
- Only studies with cancer patients in a prehabilitative context
- Reporting/availability of complete pre- and post-interventional VO_2peak_ data

#### Exclusion criteria

- Any non-interval exercise intervention trials
- Retrospective trials, case reports/case series, reviews, letters, editorials, commentary
- Language limitations (not in English or German)
- Animal studies

### Search strategy

Databases were screened for accessible English language-randomized and non-randomized controlled trials published up to March 2020. The following search terms related to cancer, exercise, and prehabilitation were used: “high intensity training” (all fields) OR “high intensity exercise” (all fields) OR “vigorous exercise” (all fields) OR “high intensity intermittent exercise” (all fields) OR “high intensity functional training” (all fields) OR “interval exercise training” (all fields) OR “interval training” (all fields) OR “high intensity interval training” (all fields) AND “prehabilitation” (all fields) OR “preoperative” (all fields) OR “supportive” (all fields) OR “surgery” (all fields).

### Study selection and data extraction

**Fig. 1:**
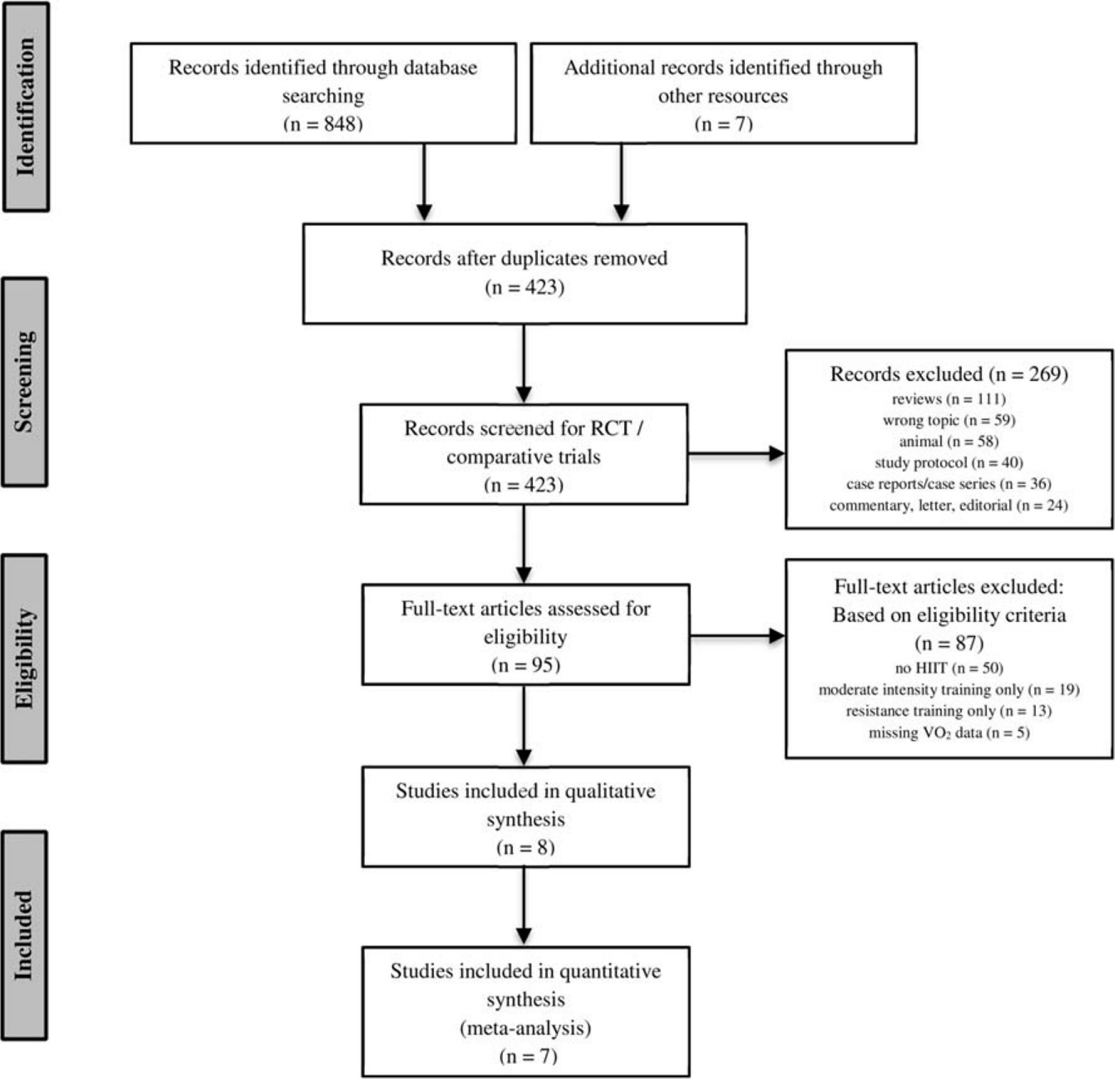
Flow chart of the screening process

Two reviewers (one medical doctor [SP] and one sport scientist [TH]) independently screened the titles and abstracts of the eligible studies. The full texts of the potentially qualified records were retrieved and screened, and relevant data were extracted. The study selection process is described in the PRISMA flow diagram (Fig. 1). In cases of insufficient data reporting, the authors of the original paper were contacted via email. If this was not possible, the study was excluded. Any discrepancies in the data extraction process between the two reviewers were resolved by discussion.

### Methodological quality assessment

To evaluate the methodological quality and risk of bias of the included studies, two reviewers independently used the 10-point PEDro scale, which is based on the Delphi list, developed by Verhagen and colleagues [33]. The PEDro scale allows a maximum of 10 points. As blinding of therapists and patients is de facto impossible to achieve in exercise intervention trials, PEDro scores of 7–8 were considered as high methodological quality. A cut-off of 5 points (equal to or greater than) on the PEDro scale indicates good quality, and a score equal to or less than 4 points indicates poor quality. Disagreements were resolved by discussion between the reviewers, or a third independent reviewer was consulted.

### Statistical analysis

The primary endpoint of the meta-analysis was the average difference in VO_2peak_ and peak power output between the two groups: HIIT vs. UC. The measurements were assessed before and after the training intervention. Some authors reported both, the means and their standard deviations. For studies that lacked this information, the mean differences were calculated with simple subtraction (“mean_end” – “mean_bsl”). The missing standard deviations at baseline and after training were back-calculated from the confidence intervals reported in the original studies (i.e., a two-sided confidence interval for a paired sample mean difference from a normal distribution with unknown variance). Some authors reported both the average difference between HIIT and UC and their confidence intervals and *p* values. For studies that were lacking this information, mean differences were calculated with simple subtraction (“mean_diff” of HIIT –“mean_diff” of UC). The 95% CI of the average difference for Bhatia et al. [23] was calculated under the assumption that the data were normally distributed (which is appropriate given the sample size). Other missing standard errors were estimated using the average correlation of two studies: Dunne et al. [34] and West et al. [35], because these studies used simple between-group comparisons, whereas other studies conducted more complex statistical analyses [17, 36], which do not allow for the calculation of the correlation based on the reported summary statistics. The statistical analyses were calculated using meta-analyses with a random intercept for each study. The models were fitted via restricted maximum-likelihood (“REML”) estimation; test statistics and confidence intervals for the fixed effects were computed based on a *t* distribution. All statistical analyses were performed using the Metafor package, R (version 3.6).

The presentation of intervention data was based on the FITT (frequency, intensity, time, and type) principle, which is an established framework for exercise prescriptions. The four components constitute the exercise quantity and dose necessary to improve health parameters equally to a pharmacologic intervention [37].

## Results

### Literature search

The combined database search yielded 855 results. After removing duplicates and screening according to inclusion criteria, eight articles fulfilled the eligibility criteria and were used for the qualitative and quantitative synthesis. The flowchart summarizes the screening process (Fig. 1).

### Overall study characteristics

The overview and characteristics of the included studies are presented in Table 1. Overall, seven RCTs [15–17, 23, 24, 34, 36] and one non-randomized controlled trial [35] with a total of 896 patients with a mean age of 61 (± 8) years were identified. Seven of the eight studies used a two-arm design (HIIT vs. UC) [16, 17, 23, 24, 34–36], whereas one study had a mixed three-arm design comparing HIIT/RT vs. HIIT/MICT vs. UC [15]. No studies were identified that compared HIIT vs. MICT in a prehabilitative setting. Three studies reported on non-small cell lung carcinoma [17, 23, 24], two on colorectal cancer [34, 35], one on breast cancer [15], one on bladder cancer [36], and one on different cancer entities [16]. Five studies were conducted on patients awaiting cancer surgery [23, 24, 34–36], two on patients during concomitant chemotherapy [15, 16], and one on patients scheduled for radiotherapy [17].

**Table 1:** Main characteristics of the included studies

Reference	Design	Patients at baseline	Age	Disease/prehabilitation phase	Inclusion/exclusion criteria	Main outcome parameters/questionnaires	Conclusion
Bhatia C, 2019 [23]	Randomized controlled trial	151	HIIT: 64 (13) UC: 64 (10)	Non-small cell lung carcinoma, stage; presurgical	Incl.: NSCLC, stage IIIA or less Excl.: contraindications for cardiopulmonary exercise testing (e.g., uncontrolled cardiac disease, severe pulmonary hypertension, uncontrolled asthma), limitations to adhere to prehabilitation (e.g., cycling difficulties)	Cardiorespiratory fitness (VO_2peak_), 6MWT, oxygen saturation, leg fatigue, and dyspnea (BORG scale)	Short-term HIIT was feasible and safe in preoperative setting and increased cardiorespiratory fitness
Egegaard T, 2019 [17]	Randomized controlled trial	13	HIIT: 64 (5.8) UC: 65 (4.7)	Non-small cell lung carcinoma; during concomitant radiotherapy	Incl.: NSCLC, age ≥ 18 years, stage IIIA-IV, WHO performance status 0–1 with concomitant chemoradiotherapy Excl.: any symptoms or circumstances that advise against physical activity; symptomatic heart disease (e.g., arrhythmia or myocardial infarction within the last 3 months)	Cardiorespiratory fitness (VO_2peak,_ WR_peak_), activity data (steps), pulmonary function, HADS, FACT-L, 6MWT, IPAQ-L	High intensity was feasible, safe, and well tolerated during concomitant chemoradiotherapy; no significant differences within or between groups in any secondary outcome
Mijwel S, 2019 [15]	Randomized controlled trial	175	HIIT/RT: 52.7 (10.3) HIIT/MICT: 54.4 (10.3) UC: 52.6 (10.2)	Breast cancer; during concomitant chemotherapy	Incl.: women with breast cancer, aged 18–70 years, stage I–IIIa, planned to receive adjuvant chemotherapy Excl.: advanced disease, heart or lung disease, cognitive dysfunction	Cardiorespiratory fitness (VO_2peak_), PFS, EORTC-QLQ C30, MSAS, muscle strength, return to work	Intervention groups showed beneficial effects on cancer-related fatigue, symptoms, and muscle strength, 12 months following the commencement of chemotherapy
Banerjee S, 2017 [36]	Randomized controlled trial	60	HIIT: 71.6 (6.8) UC: 72.5 (8.4)	Bladder cancer; presurgical	Incl.: bladder cancer patients listed for radical cystectomy (± neoadjuvant chemotherapy) Excl.: patients with urinary diversion for benign disease, patients meeting current physical activity guidelines (≥ 150 min of moderate intensity per week)	Cardiorespiratory fitness (VO_2peak_, AT, WR_peak_), feasibility, Clavien Dindo classification, LOS	HIIT was feasible and well tolerated and improved cardiopulmonary fitness
Karenovics W, 2017 [24]	Randomized controlled trial	151	HIIT: 64 (13) UC: 64 (10)	Non-small cell lung carcinoma; presurgical	Incl.: proven or suspected NSCLC, stage IIIA or less, awaiting lung resection surgery Excl.: any contraindication for CPET (e.g., uncontrolled cardiac disease, severe pulmonary hypertension, limitations impeding cycling); inability to adhere to a rehabilitation program	cardiorespiratory fitness (VO_2peak_, WR_peak_), pulmonary function test, survival (1y FU)	Preoperative rehabilitation with HIIT does not improve pulmonary function and aerobic capacity 1 year after lung cancer resection, survival after 1 year was equal, postop pulmonary complications less in HIIT
Dunne DFJ, 2016 [34]	Randomized controlled trial	38	HIIT: 61 (56–68) UC: 62 (53–72)	Colorectal liver metastasis patients, presurgical	Incl.: resectable colorectal liver metastasis, age ≥ 18 years, partake in cycle-based exercise, complete the exercise program before the proposed surgery date, at least 4 weeks of prehabilitation Excl.: pre-existing chronic liver disease, recruitment to the study must not result in delayed surgical care	cardiorespiratory fitness (VO_2peak_, AT, WR_peak_), SF-36	HIIT was feasible and safe, the intervention reduced fatigue, improved vitality, aerobic capacity, muscular strength, physical and functional activity, emotional well-being, but not quality of life
West MA, 2015 [35]	Non-randomized controlled trial	39	HIIT: 64 (45–82) UC: 72 (62–84)	Non-metastatic locally advanced rectal cancer, presurgical	Incl.: locally advanced resectable rectal cancer, age ≥ 18 years, stage T2/N+, no distant metastasis, WHO performance status < 2, undergoing NACRT Excl.: nonresectable disease, inability to perform CPET or bicycle exercise, patients who declined surgery or NACRT, patients who received non-standard NACRT	Cardiorespiratory fitness (VO_2peak_, LT, WR_peak_), spirometry, MRI staging	Chemoradiotherapy before rectal cancer surgery reduced physical fitness; however, a 6-week exercise intervention was feasible and returns fitness to baseline levels
Adamsen L, 2009 [16]	Randomized controlled trial	269	HIIT: 47.2 (10.7) UC: 47.2 (10.6)	Non-metastatic cancer patients undergoing chemotherapy	Incl.: a diagnosis of cancer, aged 18–65 years, at least one cycle of chemotherapy for advanced disease or as adjuvant treatment, WHO performance status of 0 or 1 Excl.: brain or bone metastases, thrombocytopenia (< 50 × 109/l), myocardial infarction within the past 3 months, uncontrolled hypertension (diastolic pressure > 95 mmHg)	Cardiorespiratory fitness (VO_2peak_), EORTC-QLQ C30, Medical Outcomes, SF-36, Leisure Time Physical Activity Quest., muscular strength	HIIT was feasible and safe, the intervention reduced fatigue, improved vitality, aerobic capacity, muscular strength, physical and functional activity, emotional well-being, but not quality of life

*Incl* inclusion, *Excl* exclusion, *BL* baseline, *FU* follow-up, *AT* anaerobic threshold, *LT* lactate threshold, *WRpeak* work rate peak, *EORTC-QLQ C30*, European Organisation for Research and Treatment of Cancer/Core Quality of Life Questionnaire, *LOS* length of stay, *PFS* Piper Fatigue Scale, *MSAS* Memorial Symptom Assessment Scale, *HADS* Hospital Anxiety and Depression Scale, *IPAQ-L* International Physical Activity Questionnaire, *NSCLC* non-small cell lung carcinoma, *CPET* cardiopulmonary exercise testing, *NACRT* neoadjuvant chemoradiotherapy

Concerning eligibility criteria, five studies included patients with a diseases stage of III or less (= without peripheral metastases) [15, 16, 23, 24, 35], two studies explicitly included more advanced stages of disease [17, 34], whereas one study did not report any staging [36]. To determine pre-treatment performance status of cancer patients, three studies limited the inclusion criteria for patients to a WHO performance status < 1 [16, 17] and < 2 [35], while the other studies did not mention any criteria.

One of the eight studies assessed exercise history and consequently excluded patients who performed more than 150 min of moderate intensity per week [36]. Overall physical activity levels were assessed in two studies with the International Physical Activity Questionnaires [17] and Leisure Time Physical Activity Questionnaire [16].

### Quality assessment

Table 2 summarizes the quality of the included studies. The total scores for methodological quality ranged from 5 to 8 points on the 10-point PEDro scale. Four studies presented a high methodological quality [16, 23, 24, 36], and four studies presented a good methodological quality [15, 17, 34, 35].

**Table 2:** Assessment of the quality of the included studies

First author	Eligibility criteria*	Random allocation	Concealed allocation	Groups similar at baseline	Blinded subjects	Blinded therapist	Blinded assessors	> 85% key outcomes of subjects obtained	Intention-to-treat analysis	Between-group differences	Point and variability	Total
Bhatia C, 2019 [23]	(1)	1	1	1	-	-	1	1	1	1	1	8
Egegaard T, 2019 [17]	(1)	1	1	1	-	-	-	1	-	1	1	6
Mijwel S, 2019 [15]	(1)	1	-	1	-	-	-	1	1	1	1	6
Banerjee S, 2017 [36]	(1)	1	1	1	-	-	1	1	-	1	1	7
Karenovics W, 2017 [24]	(1)	1	1	1	-	-	1	1	1	1	1	8
Dunne DFJ, 2016 [34]	(1)	1	-	1	-	-	1	1	-	1	1	6
West MA, 2015 [35]	(1)	-	-	-	-	-	1	1	1	1	1	5
Adamsen L, 2009 [16]	(1)	1	1	1	-	-	-	1	1	1	1	7

^*^Eligibility criteria not counted for total score

### Intervention characteristics

The main characteristics of the exercise interventions are presented in Table 3 according to the FITT principle. The frequency of a single exercise session was presented in all studies and ranged from two [15, 36] to five times per week [17]. Intensity prescription was reported in all studies except for one [15], whereas the specific interval configuration was accurately shown in all trials except for two [16, 34]. The duration of a single high-intensity bout lasted from 15 s [23, 24] to 5 min [36]. The length of a HIIT intervention per session (without warm up and cool down phases) lasted from 11 [15] to 40 min [36], and the sessions were integrated into total intervention periods lasting between 2 and 3 [23] and 16 weeks [15]. The progression of exercise based on individualized intensity prescription with cardiopulmonary exercise testing (CPET) at baseline was reported in all studies. The prescribed intensity was defined as a given percentage of peak work rate [17, 23], peak oxygen consumption [34, 35], and peak heart rate [16, 36] achieved at the CPET. Furthermore, three of the eight studies used the Borg scale of perceived exertion to additionally adjust for intensity [17, 23, 36]. All interventions were supervised and executed on a cycle ergometer. Two studies performed long-term follow-up lasting more than 1 year [15, 24]. The adherence of the study population was apparent in six of the eight studies [16, 17, 23, 24, 35, 36] and ranged from 71 [16] to 96% [35]. One study had a multimodal prehabilitative approach that included relaxation, body awareness training, and massage in the exercise training program [16]. Survival data were presented in one study, with a similar distribution of deaths reported after a 1-year follow-up in the study population [24].

**Table 3:** Characteristics of the exercise interventions

Reference	*n*	Study arm	Frequency	Intensity	Time (duration per session, only HIIT)	Type	Intervention period	Timepoints	Adherence/attendance
Bhatia C, 2019 [23]	151	HIIT	3×/week	5 min WU (50%WR_peak_) HIIT: 2 × 10 × 15 s (100%WR_peak_)/15 s passive rest, 4 min pause 5 min CD (30% W_peak_)	20 min	Cycle ergometer	2–3 weeks	BL preoperative	Adherence 87% (± 18%)
		UC	Usual care						
Egegaard T, 2019 [17]	13	HIIT	5×/week	5 min WU (50–60% WR_peak_) HIIT: 2 × 5 × 30 s (80–95% WR_peak_)/30 s pause - 5 min continuous at 80% WR_peak_ 3 min CD (40%VO_2peak_)	20 min	Cycle ergometer	7 weeks	BL pre-radiotherapy	Attendance 90.0% (53.8–100%), adherence 88.1% (70.0–100.0%)
		UC	Usual care, wearing activity tracker						
Mijwel S, 2019 [15]	175	HIIT + RT	2×/week	RT: 2–3 × 8–12 repetitions (70 to 80% 1-RM) HIIT: 3 × 3 min HIIT/1 min recovery	11 min	Cycle ergometer	16 weeks	BL 16 weeks 1 year follow-up	Attendance 68%
		HIIT + MICT	2×/week	HIIT: 3 × 3 min /1 min recovery MICT: 20 min	11 min (HIIT), 20 min (MICT)	Cycle ergometer	16 weeks		Attendance 63%
		UC	Usual care, exercise recommendation for cancer patients given according to ACSM						
Banerjee S, 2017 [36]	60	HIIT	2×/week	5–10 min WU (50 W), 6 × 5 min HIIT (target BORG 13–15, equating to 85–87% HR_max_ based on 220-age and 90–92% on HR_peak_ on CPET), 2.5 min interpolated active rest (50 W), CD (50 W)	40 min	Cycle ergometer	3–6 weeks	BL preoperative	Adherence 90% Attendance 8 sessions (1–10)
		UC	Usual care, maintain usual lifestyle				29 days		
Karenovics W, 2017 [24]	151	HIIT	3×/week	5 min WU (50% WR_peak_) HIIT: 15 s sprint intervals (“all-out” effort) interspersed by 15 s pauses, 4 min rest, 5 min CD	20 min	Cycle ergometer	2–3 weeks	BL preoperative 1 year follow-up	Adherence 87 ± 18% (median 8 sessions, IQ25–75% [7–10])
		UC	Usual care						
Dunne DFJ, 2016 [34]	38	HIIT	3×/week	WU, HIIT (alternating between moderate (less than 60% VO_2peak_) and vigorous (more than 90% VO_2peak_) intensity, CD	30 min	Cycle ergometer	4 weeks	BL preoperative	Attendance 95% completed 100% of sessions
		UC	Usual care, general recommendations on exercise before surgery						
West MA, 2015 [35]	39	HIIT	3×/week	5 min WU, 4 × 3 min to 6 × 3 min (moderate intensity at 80% of work rate at VO_2_ at LT) and 4 × 2 min to 6 × 2 min (severe intensity at 50% of the difference in work rates between VO_2peak_ and VO_2_ at LT	30 min	Cycle ergometer	6 weeks	BL pre-chemoradio post-chemoradio week 6, 9, 14	Adherence 96% (5.3)
		UC	Usual care						
Adamsen L, 2009 [16]	269	HIIT, RT, psychosocial	3×/week	30 min WU RT: 45 min (3 × 5–8 repetitions at 70 to 100% 1-RM) HIIT: 15 min (workload 70-250 W, equivalent to 85–95% of HR_max_)	15 min	Cycle ergometer	6 weeks	BL pre-chemotherapy	Adherence 70.8% (17 of 24 training days, range 3–24)
		UC	Usual care; are allowed to freely increase physical activity						

*HIIT* high-intensity interval training, *UC* usual care, *RT* resistance training, *WU* warm up, *CD* cool down, *BL* baseline, *W* watts, *WRpeak* work rate peak, *LT* lactate threshold, *1-RM* one repetition maximum

Five of the eight studies reported no adverse events [17, 23, 34–36], and one study reported three drop-outs during the cardiovascular training. One patient with a brain tumor was excluded due to a seizure episode, and two patients withdrew from the study because of leucopenia and/or increased blood pressure [16].

### Descriptive analyses

The mean values and standard deviations at baseline and at the end are presented in Supp. Tables 1 and 2.

### Meta-analysis peak oxygen uptake (VO_2_max)

**Fig. 2:**
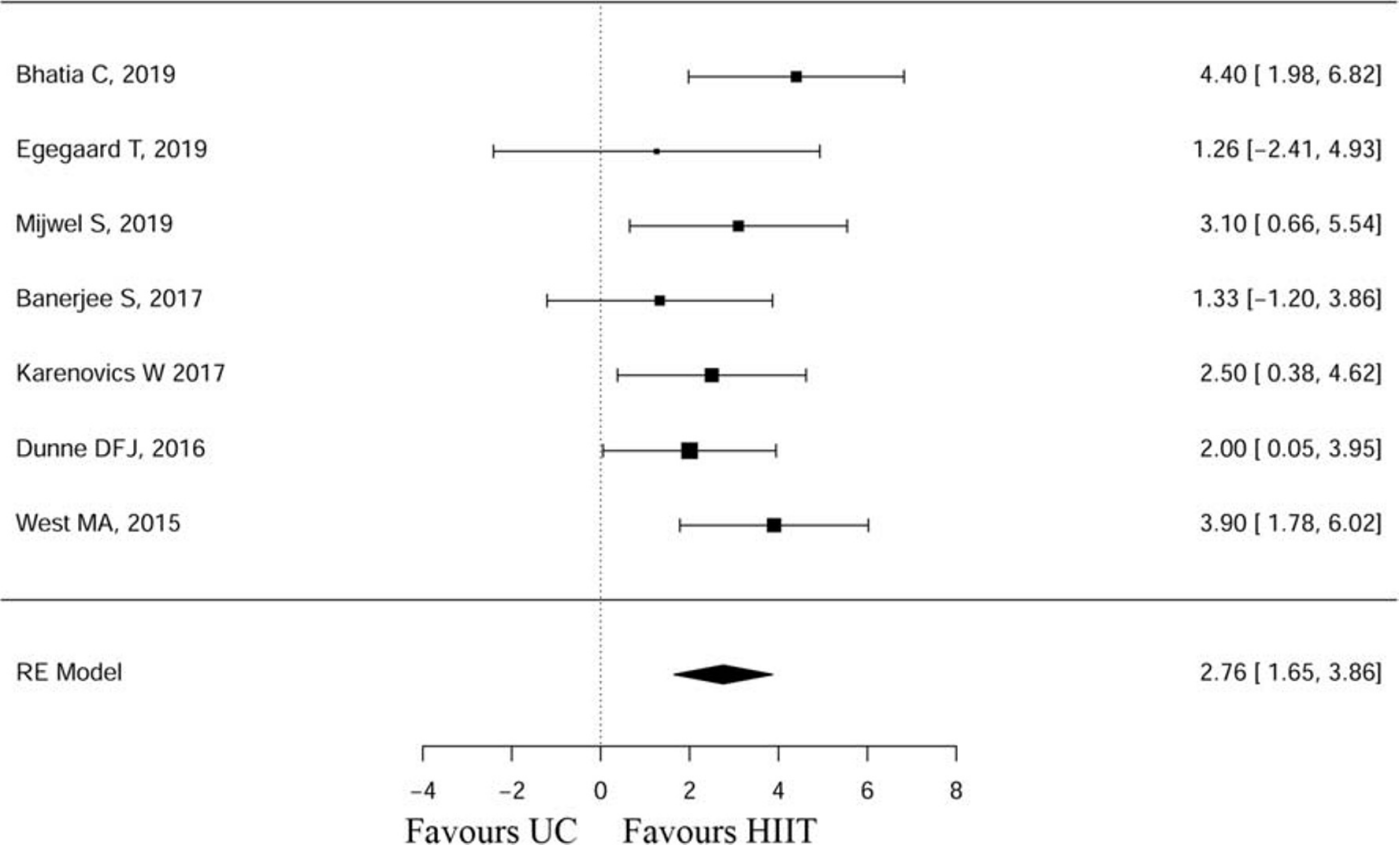
Forest plot of peak oxygen uptake (ml kg^−1^ min^−1^)

Figure 2 shows an overview of the effects of the HIIT intervention compared to UC (MD 2.76, 95% CI 1.65, 3.86). The heterogeneity test results were as follows: Q(df = 6) = 5.45, *p* = 0.4872. Publication bias for VO_2max_ was assessed with a funnel plot graph (Supp. Figure 1).

### Meta-analysis peak work rate

**Fig. 3:**
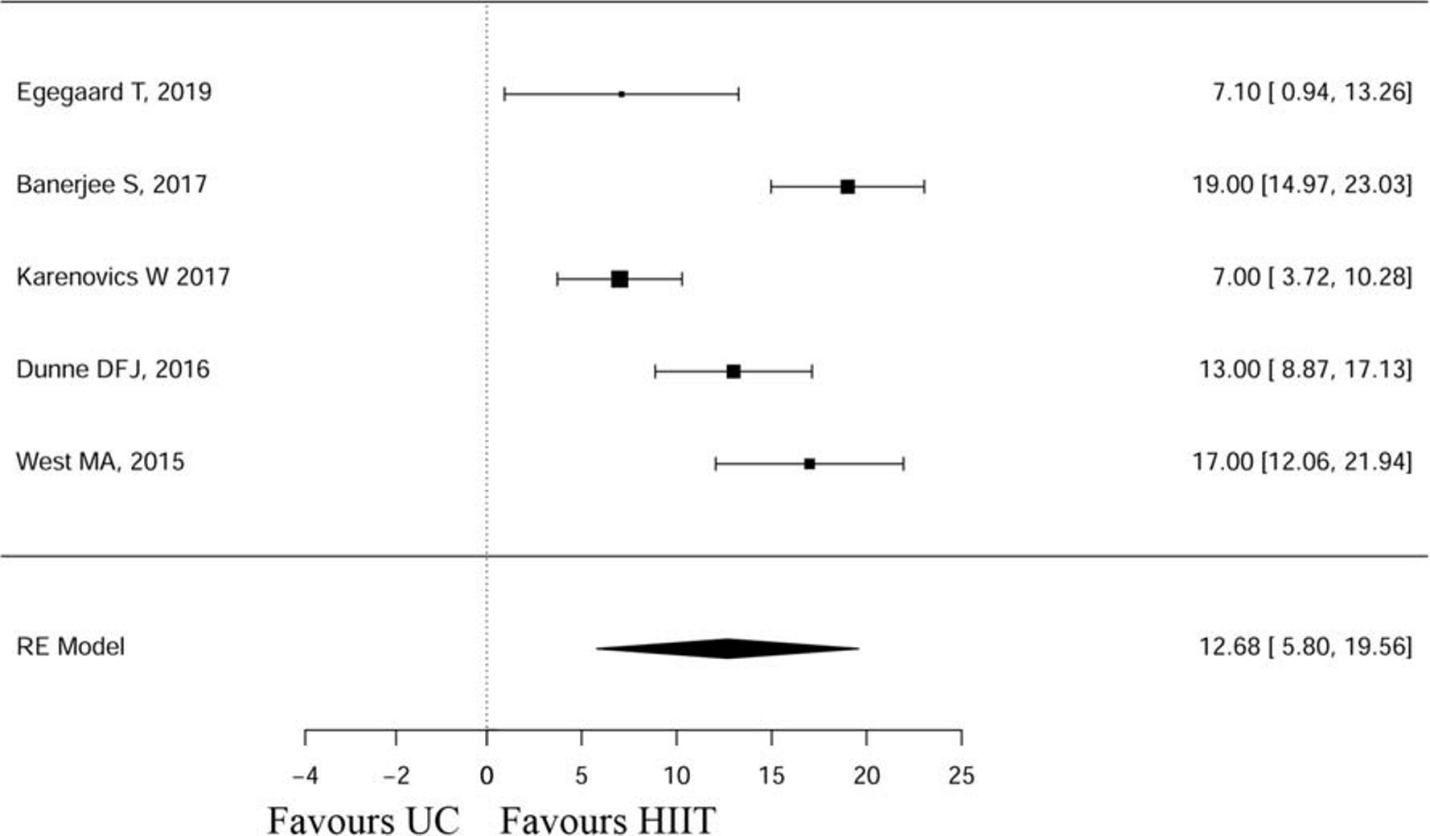
Forest plot of the peak work rate (watts)

Figure 3 illustrates an overview of the effects of the HIIT interventions compared with UC on the peak work rate (MD 12.68, 95% CI 5.80, 19.56). The heterogeneity test results were as follows: Q(df = 4) = 26.96, *p* < .0001. Publication bias for the peak work rate is illustrated in Supp. Figure 2.

### Functional outcomes

Two studies each reported functional capacity outcomes such as the 6MWT [17, 23], with significant improvements in one trial (median 20%, 95% CI 14–26%, *p* < 0.001) [23]; in studies testing muscle strength, gains were reported in lower limb strength (effect size 1.03), handgrip strength (effect size 0.59–0.71) [15], and an average growth of 29.6% (SD 36.4) for leg press, chest press, and pull down in the treatment arm compared with UC [16]. Pulmonary function was assessed in two studies, but no significant differences were shown between baseline and post-intervention in the two groups [17, 24].

### Patient-reported outcomes

Health-related QoL was evaluated in five of the eight studies. Two studies evaluated this factor with the European Organization for Research and Treatment of Cancer questionnaire (EORTC-QLQ C30) [15, 16], two studies used the 36-item short form (SF-36) [16, 34], and one study used the Functional Assessment of Cancer Therapy—Lung survey (FACT-L) [17]. One study noted significant effects on the fatigue subscale (effect size 0.33, *p* < 0.05) of the EORTC-QLQ C30 [16], whereas two studies found significant effects in several subscales of the SF-36, with the results favoring the HIIT intervention [16, 34].

Over a 1-year follow-up period, one study demonstrated a significant reduction in cancer-related fatigue (effect size − 0.34, *p* = 0.012), assessed by the Piper Fatigue Scale (PFS); additionally, in their assessment of return to work (RTW), they revealed that the proportion of patients on more than half-time sick leave was significantly lower in the MICT/HIIT group compared with patients receiving UC (5.9 vs. 31%, *p* = 0.006). The same study noted that the symptom burden measured with the Memorial Symptom Assessment Scale (MSAS) was significantly lower in both treatment arms (effect size − 0.46) after 12 months of follow-up [15].

One study assessed psychosocial parameters such as depression and anxiety indicators with the Hospital Anxiety and Depression Scale (HADS) and demonstrated no significant differences within or between groups in the study population [17].

Two of the eight studies reported on pre-interventional physical activity (PA) with self-report instruments such as the Leisure Time Activity Questionnaire [16] and the revised version of the International Physical Activity Questionnaire (IPAQ-L) [17].

### Additional clinical outcomes

Activity data (steps) were tracked with an accelerometer in two studies [17, 35]. One study showed a significant difference in the within-group comparison of the average number of steps for the exercise (*p* < 0.0001) and control groups (*p* = 0.003) during the 6-week intervention. However, between-group differences were not significant in this study (*p* = 0.84) [35].

Tumor regression was assessed and classified in one study with magnet resonance imaging (MRI), revealing a significant clinical response to the applied neoadjuvant chemoradiotherapy in favor of the exercise intervention group (*p* = 0.006) [35].

In the five studies in which HIIT preceded cancer surgery [23, 24, 34–36], three studies reported on postoperative recovery outcomes such as length of stay in the post-anesthesia care unit/hospital and postoperative complications [24, 34, 36]. One of these studies reported a significant difference in the study groups for postoperative outcomes. Karenovic et al. demonstrated that prehabilitation led to a reduction in pulmonary complications (23% in the HIIT group vs. 44% in the UC group, *p* = 0.018) and a shorter stay in the post-anesthesia care unit (median − 7 h, interquartile range, − 4 to − 10 h) in lung cancer patients [24].

## Discussion

This systematic review and meta-analysis investigated the effects of HIIT in the prehabilitation of cancer patients. Recently, a shift from a reactive curative to a proactive preventive approach in healthcare is emerging, with a primary focus on optimization of patients’ health status between diagnosis and post treatment recovery. During preoperative care, prehabilitative interventions such as exercise have been shown to accelerate recovery by mitigating potential treatment-related impairments and deconditioning [11, 13]. In that regard, HIIT emerged as an effective and time-efficient aerobic exercise modality to gain maximal aerobic capacity in a relatively short time period [23]. However, evidence about its implementation in prehabilitative settings is scarce, and reviews focusing solely on prehabilitation and HIIT are missing.

This systematic review and meta-analysis of seven included trials revealed a significant increase in aerobic capacity due to HIIT programs (reflected by VO_2max_ growth, which is a strong singular predictor of cancer-related mortality) compared with UC. This finding is consistent with a study reporting on HIIT interventions to increase VO_2max_ in the therapy and aftercare of cancer patients [31]. Furthermore, despite the heterogeneity of the study designs and their small sample sizes, the descriptive data analysis demonstrated that supervised HIIT is a feasible and safe training method for prehabilitation, with a low risk of major adverse events. Moreover, it contains some notable health benefits for patients such as a reduction of cancer-related fatigue, increased health-related QoL, increased muscle strength, tumor regression after neoadjuvant chemoradiotherapy, and faster RTW after cancer therapy in favor of the intervention group; no significant changes in pulmonary function were observed. However, findings on other functional and clinical outcomes were inconsistent, specifically the 6MWT and postoperative outcomes such as length of stay and postoperative complications.

From eight included studies in this meta-analysis, two considered stage IV patients with peripheral metastasis, which indicated that HIIT might be safe also for patients with an advanced disease. However, this finding is difficult to extrapolate to other cancer types and definitely needs to be proven in future studies. Generally, patients with cardiovascular contraindications for CPET, such as severe cardiopulmonary limitations, were a priori excluded in most studies. One study restricted the eligible patients with a WHO performance status of < 1, but simultaneously included stage IIIA to IV patients [17], which implied that advanced disease does not necessarily need be accompanied by a low performance status. As most cancers usually occur in the elderly population, where comorbidities are common [38], prehabilitation offers an opportunity to additionally optimize modifiable pre-existing risk factors.

In summary, each patient has unique comorbidities and physical capabilities, and as a result, exercise needs to be individually tailored to meet each patient’s specific needs, based on an adequate baseline assessment and (ideally) subsequent monitoring and professional guidance during training sessions.

As discussed earlier, a preoperative, high-intensity program of interval training within a short time frame prior to surgery is supposed to increase the patient’s aerobic capacity, aiming to minimize operative stress and complications. Indeed, VO_2peak_, a strong indicator of aerobic capacity, is the best independent predictor of the surgical complication rate [39]. Therefore, it appears logical to evaluate the efficacy and clinical impact of prehabilitation programs by assessing potential postoperative complications and the length of hospital stay. Three of the eight studies included in this review presented postoperative outcomes; however, only one study reported a significant reduction in (pulmonary) complications and a shorter stay in the post-anesthesia care unit [24]. To increase the relevance of HIIT interventions in prehabilitation, future research should put a stronger focus on postoperative outcomes.

A substantial number of studies reported associations between regular physical activity and all-cause mortality [40–42], thus indicating physical activity to be an independent risk factor for specific chronic diseases, including cardiovascular diseases, diabetes, and colon and breast cancer [43, 44]. However, to date, the underlying dose-response relationship remains unclear. Following international guidelines, strong evidence for the beneficial effects of exercise on health-related outcomes exists, including anxiety, depressive symptoms, QoL, physical function, and lymphedema. Depending on the outcome, a target intensity for aerobic exercise between 60 and 80% of maximum heart rate (HR_max_), which corresponds to 60 to 80% of VO_2max_ with a duration of 30 to 60 min, is recommended [8]. Presuming that a one-size-fits-all approach cannot be applicable for all patients, the specification of the individualized target intensity zones varies between experts, using different models, i.e., percentages of VO_2peak_, heart rate reserve/max, or work rate peak after CPET before an intervention starts [8, 45]. In a critical analysis of these general exercise prescription recommendations, applied after completing primary therapy, breast cancer survivors tended to either be overloaded or under-challenged when using percentages of heart rate reserve or VO_2max_; conversely, HR_max_ appeared to be adequately intense for this specific population [46]. In this context, individualized threshold concepts, i.e., blood lactate or ventilatory thresholds (as a gold standard), are considered more accurate and reflect an individualized metabolic profile [47]. The studies included in this review used a heterogeneous approach of prescribing individualized workload intensities and included WR_peak_, HR_peak_, and VO_2peak_, without explaining how interval variables (duration, frequency, or intensity) were selected, and failed to account for differences in cardiovascular and metabolic stress. No study considered these threshold concepts in their methods, except one study which applied a mixed model with lactate threshold and VO_2peak_ for vigorous intensity [35]. This might be of particular relevance for cancer patients, as a recent meta-analysis concluded that HIIT was more efficient than continuous training in patients with coronary artery disease at increasing both VO_2peak_ and the anaerobic threshold (*p* < 0.01 for each) [48].

Optimizing exercise-related outcomes is the present challenge in the prehabilitation of cancer patients; as over the past decades, cancer management has substantially improved, and modern therapeutic strategies in cancer are associated with higher survival rates [49]. It is often overlooked that approximately 40% of cancer survivors are < 65 years old; hence, they are potentially of an employable age, but long-term symptoms and impairments, such as fatigue or physical weakness, might threaten cancer patients’ daily life activity. RTW after sick leave is challenging, and an observed higher unemployment rate of cancer patients is often accompanied by social isolation, financial losses, and reductions in self-esteem [50, 51]. In this review, one study discussed aspects of sick leave and RTW and reported a significantly lower proportion of patients on more than half-time sick leave in the intervention group [15]. Optimizing the prehabilitation and RTW of workers with cancer is important for improving the well-being of this vulnerable group and for reducing the societal and financial impacts of cancer.

This review and meta-analysis have some limitations. Given the high degree of data variability and heterogeneity of the included studies, suitable data was only available for two outcome parameters (VO_2peak_ and work rate peak). Furthermore, the number of studies and sample sizes was low. However, no publication bias was indicated in the funnel plots. No three-arm designs (moderate continuous intensity training vs. HIIT vs. UC) were identified during the screening process; therefore, no respective comparative analysis was executable.

These limitations highlight the present deficits in research. For a comprehensive understanding of the effects of HIIT in cancer prehabilitation, future research needs to focus on high-quality comparative studies between HIIT and MICT, and encompass exercise planning via threshold concept vs. established methods, in various cancer entities, stages, and therapy regimes. Moreover, not just the effects of HIIT on performance parameters but also on postoperative complication rates, long-term survival, and socio-economic impact urgently need to be assessed. All of these puzzle pieces are required for the ultimate aim of prescribing individualized exercise recommendations for each single cancer patient.

## Conclusion

HIIT is a novel and pragmatic exercise method in prehabilitation, and this meta-analysis showed that it displays significant benefits on aerobic capacity and peak power output compared with UC, despite the short intervention duration applied in some studies.

## Electronic supplementary material

ESM 1 (PNG 64 kb)

High resolution image (TIF 9055 kb)

ESM 2 (PNG 64 kb)

High resolution image (TIF 9101 kb)

ESM 3 (DOCX 14 kb)

ESM 4 (DOCX 13 kb)
