# Supplementary figures and images for: High-intensity interval training in the prehabilitation of cancer patients—a systematic review and meta-analysis
Source: Support Care Cancer. 2020 Oct 26;29(4):1781–94. doi: 10.1007/s00520-020-05834-x (PMC7892520; doi:10.1007/s00520-020-05834-x)

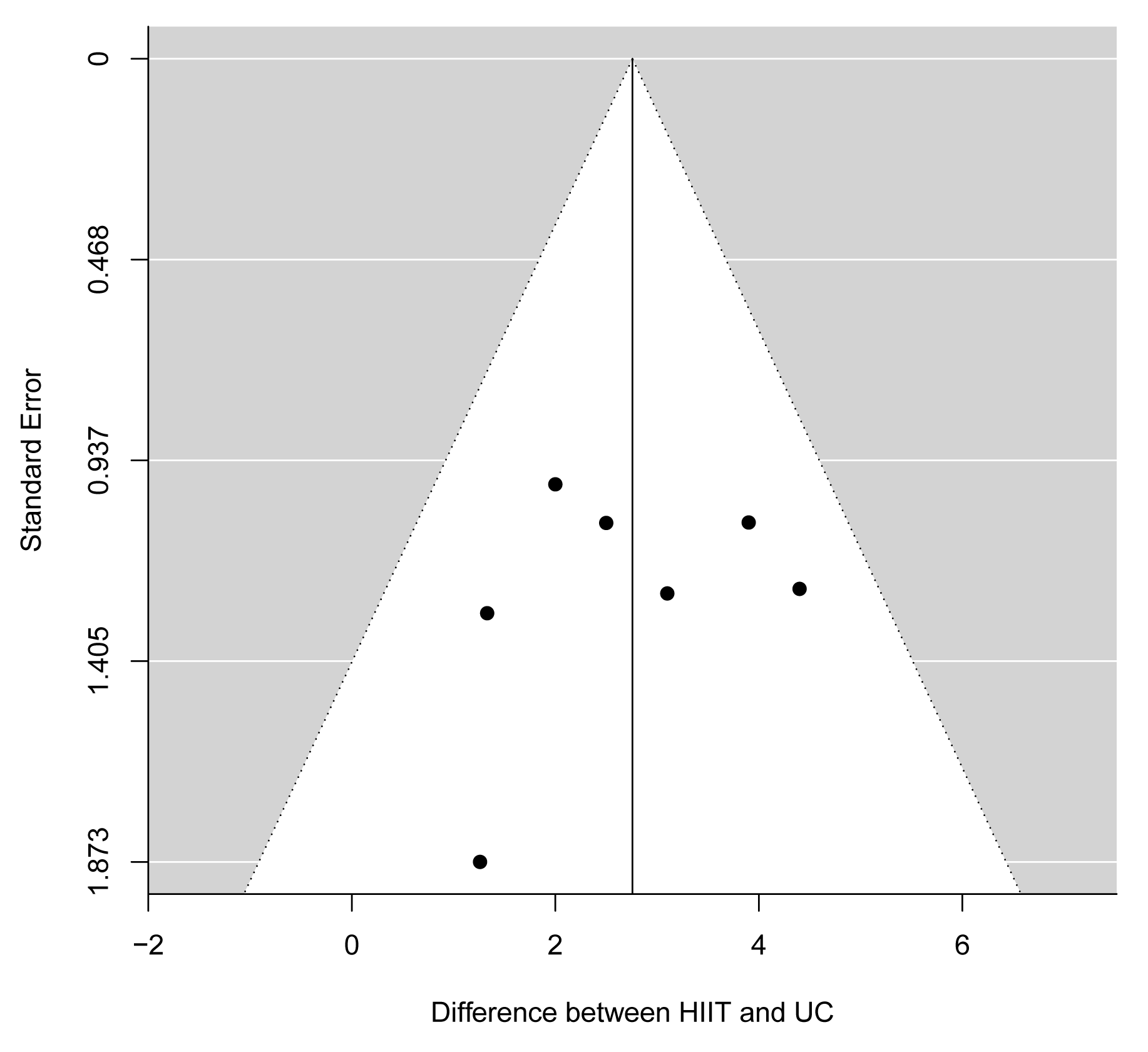

Supplement: Supplementary file 1 — (PNG 64 kb) [file 520_2020_5834_Fig4_ESM.png]

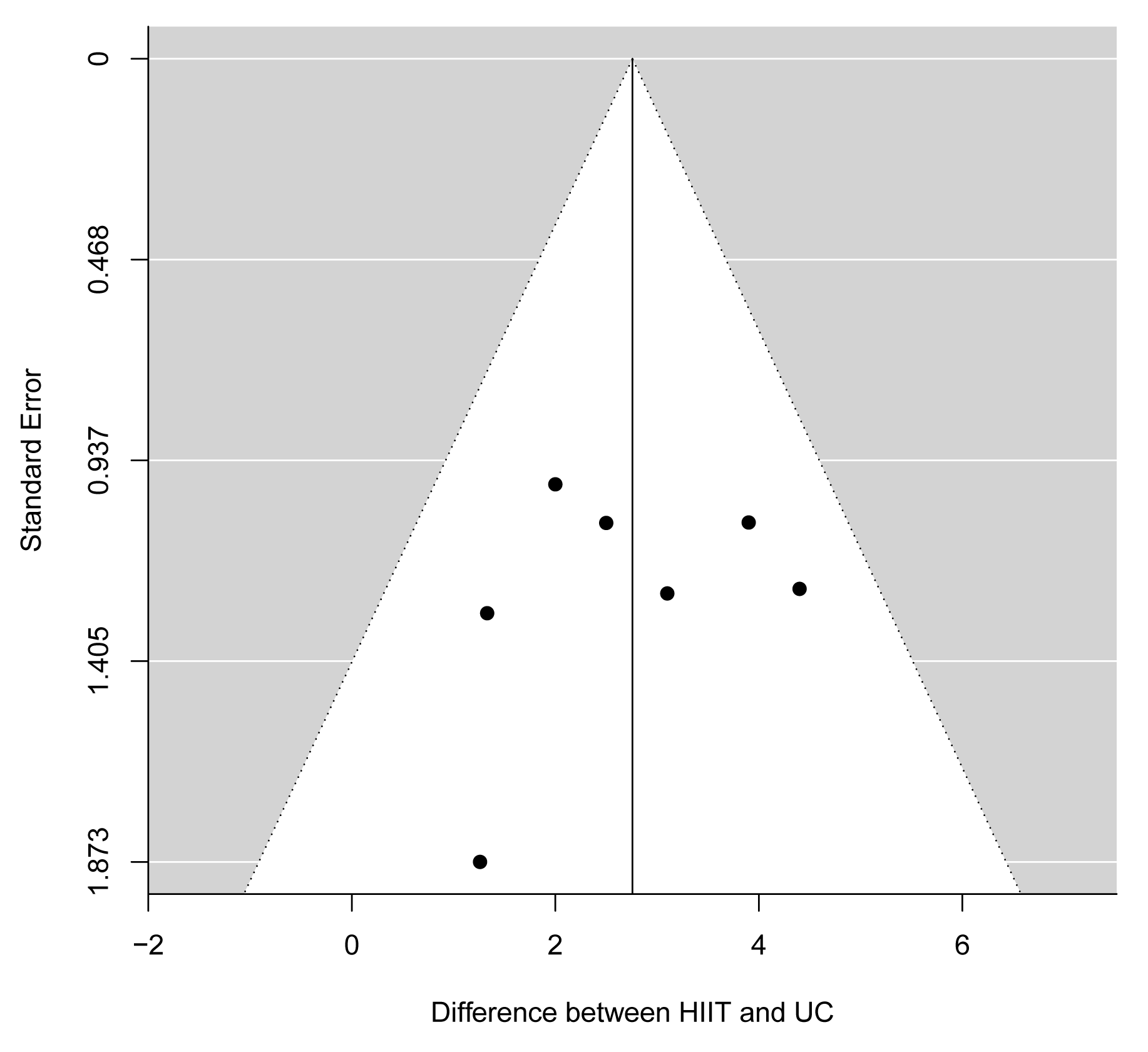

Supplement: Supplementary file 2 — High resolution image (TIF 9055 kb) [file 520_2020_5834_MOESM1_ESM.tif]

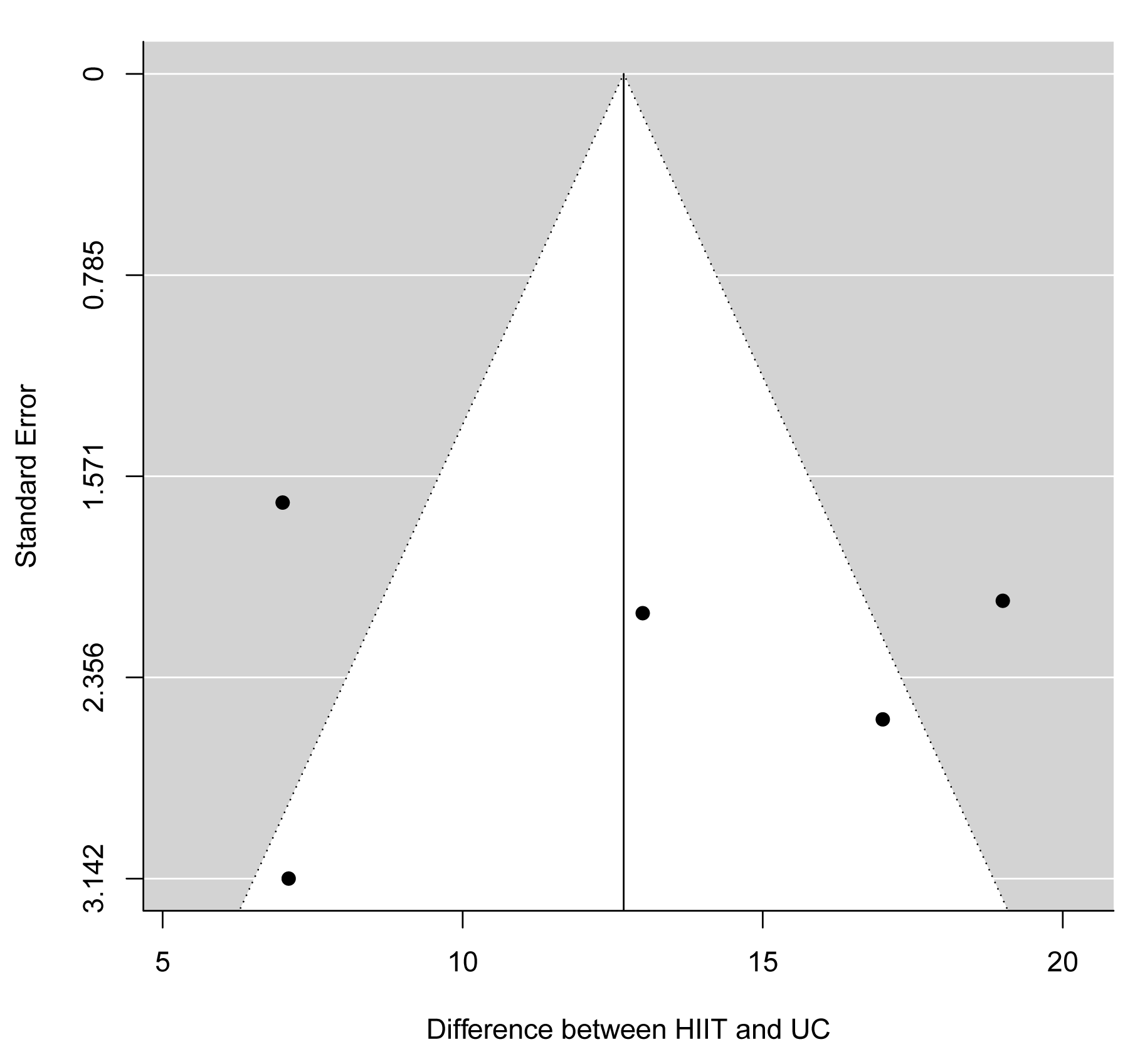

Supplement: Supplementary file 3 — (PNG 64 kb) [file 520_2020_5834_Fig5_ESM.png]

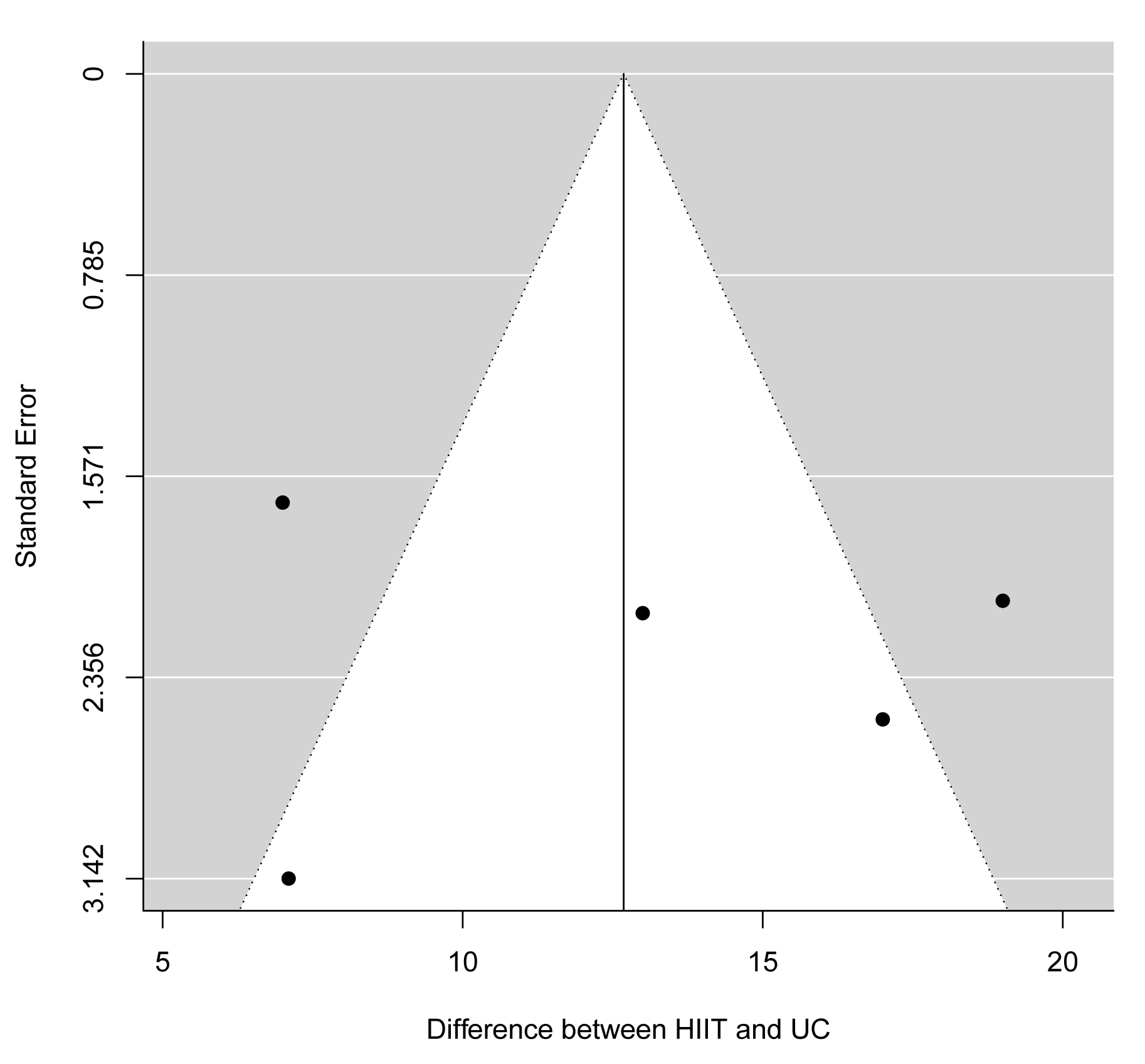

Supplement: Supplementary file 4 — High resolution image (TIF 9101 kb) [file 520_2020_5834_MOESM2_ESM.tif]
