# Supplementary material for: High-intensity interval training in the prehabilitation of cancer patients—a systematic review and meta-analysis
Source: Support Care Cancer. 2020 Oct 26;29(4):1781–94. doi: 10.1007/s00520-020-05834-x (PMC7892520; doi:10.1007/s00520-020-05834-x)
Supplement: Supplementary file 5 — (DOCX 14 kb) [file 520_2020_5834_MOESM3_ESM.docx]

**Table 4**. Peak oxygen uptake (ml·kg^-1^·min^-1^)

| reference | group | n | mean_bsl | sd_bsl | mean_end | sd_end |
| --- | --- | --- | --- | --- | --- | --- |
| Bhatia C, 2019 | HIIT | 74 | 19.90 | 5.70 | 22.80 | 2.30 |
|  | UC | 77 | 20.40 | 5.70 | 18.90 | 2.74 |
| Egegaard T, 2019 | HIIT | 8 | 19.50 | 3.60 | 18.70 | 2.80 |
|  | UC | 5 | 24.50 | 5.20 | 23.80 | 6.60 |
| Mijwel S, 2019 | HIIT | 65 | 33.45 | 7.91 | 31.70 | 8.26 |
|  | UC | 57 | 32.40 | 7.80 | 27.55 | 6.64 |
| Banerjee S, 2018 | HIIT | 27 | 19.22 | 4.80 | 21.07 | 5.60 |
|  | UC | 25 | 20.38 | 5.60 | 20.84 | 5.43 |
| Karenovics W, 2017 | HIIT | 74 | 19.90 | 5.61 | 21.10 | 8.20 |
|  | UC | 77 | 20.40 | 5.73 | 19.10 | 5.29 |
| Dunne DFJ, 2016 | HIIT | 20 | 17.60 | 2.30 | 19.60 | 3.80 |
|  | UC | 17 | 18.60 | 3.90 | 18.70 | 4.10 |
| West MA, 2015 | HIIT | 22 | 16.00 | 4.30 | 18.70 | 4.30 |
|  | UC | 13 | 15.70 | 5.00 | 14.40 | 4.50 |

*bsl* baseline, *sd* standart deviation
