# Supplementary material for: High-intensity interval training in the prehabilitation of cancer patients—a systematic review and meta-analysis
Source: Support Care Cancer. 2020 Oct 26;29(4):1781–94. doi: 10.1007/s00520-020-05834-x (PMC7892520; doi:10.1007/s00520-020-05834-x)
Supplement: Supplementary file 6 — (DOCX 13 kb) [file 520_2020_5834_MOESM4_ESM.docx]

**Table 5**. Peak power output (watts)

| reference | group | n | mean_bsl | sd_bsl | mean_end | sd_end |
| --- | --- | --- | --- | --- | --- | --- |
| Egegaard T, 2019 | HIIT | 8 | 90.00 | 29.00 | 93.00 | 21.00 |
|  | UC | 5 | 130.00 | 35.00 | 132.00 | 36.00 |
| Banerjee S, 2018 | HIIT | 27 | 131.00 | 39.00 | 148.00 | 41.00 |
|  | UC | 25 | 131.00 | 36.00 | 129.00 | 44.00 |
| Karenovics W, 2017 | HIIT | 74 | 96.00 | 34.53 | 102.00 | 17.27 |
|  | UC | 77 | 99.00 | 39.65 | 98.00 | 39.65 |
| Dunne DFJ, 2016 | HIIT | 20 | 125.00 | 26.00 | 138.00 | 35.00 |
|  | UC | 17 | 138.00 | 39.00 | 140.00 | 39.00 |
| West MA, 2015 | HIIT | 22 | 109.00 | 46.80 | 131.00 | 53.57 |
|  | UC | 13 | 80.00 | 33.10 | 85.00 | 20.69 |

*bsl* baseline, *sd* standart deviation
